# Multiomics analysis reveals the biochemical and genetic bases of *Coffea arabica* L. fruit color

**DOI:** 10.7717/peerj.20305

**Published:** 2026-04-28

**Authors:** Ruifang Wang, Zhengwan Xie, Gengyun Pan, Liye Yang, Liping Liu, Xiu Li, Haifei Lu

**Affiliations:** 1 School of Tea and Coffee, Pu’er University, Pu’er, China; 2 College of Urban Construction, Zhejiang Shuren University, Hangzhou, China

**Keywords:** Anthocyanin, Carotenoid, Mutiomics, Genes, *Coffea arabica* L.

## Abstract

Coffee is a remarkable source of bioactive compounds, including anthocyanins, proanthocyanidins, and carotenoids, with health benefits. To fully understand the biochemical and molecular regulation mechanisms behind *Coffea arabica* L. pigmentation, transcriptome profiling and metabolite quantitation were conducted on three varieties with significant color differences. A total of 40 anthocyanins, six proanthocyanidins, and 34 carotenoids were accumulated in three *C. arabica* fruits. The up-regulation and accumulation of most flavonoids, including anthocyanidin compounds, and the down-regulation and accumulation of proanthocyanidins were important factors in the purple pigmentation of *C. arabica* fruits, while the down-regulation and accumulation of anthocyanins and the up-regulation and accumulation of proanthocyanidins were important factors affecting the yellow pigmentation of *C. arabica* fruits. Cyanidins were major contributors to redness in the anthocyanins of *C. arabica* fruits, especially the cyanidin-3-O-glucoside compound. The accumulation of anthocyanin metabolites was the main factor affecting the purple pigmentation of *C. arabica* fruits, followed by the high proportion of cyanidin metabolites and the unique metabolites of petunidan. The accumulation of proanthocyanidins contributed to the yellow and pink pigmentation of *C. arabica* fruits, with Procyanidin B2 and Procyanidin C1 metabolites contributing specifically to yellow pigmentation. Lutein and lutein myristate were major contributors to carotenoids of *C. arabica* fruits, and lycopene and Zeaxanthin-laurate-myristate contributed to the red pigmentation of *C. arabica* fruits. *CaCHSs*, *CaCHIs*, *Ca4CLs*, and *CaF3Hs* could help enhance the synthesis of important compounds, including chalcone, naringenin, and dihydroflavonol, in the upstream pathway of anthocyanin synthesis. *CaF3′Hs* significantly promoted metabolic influx into cyanidin- and peonidin-related biosynthesis. *CaANSs* and *CaDFRs* were responsible for anthocyanin deposition in *C. arabica* fruits. *CaPSYs*, *CaPDSs*, *CaZ-ISO*, and *CaZDS2* were important for the formation of lycopene in *C. arabica* fruits. The key structural gene expression of *CaPSYs*, *CaPDSs*, *CaZ-ISO*, *CaZDS2*, *CaCHYE3*, *CaCHYE5*, *CaZEP9*, and *CaCRTISOs* in *C. arabica* fruits contributed to carotenoid biosynthesis. This study provides a robust biochemical analysis, revealing target genes of *C. arabica* fruit pigment deposition, which have important implications for improving the nutritional quality of *C. arabica* fruits *via* molecular breeding.

## Introduction

Coffee is one of the most valuable and most consumed beverages in the world and is grown worldwide, with most of its production occurring in the coffee belt (Zaman & Shan, 2024). The Yunnan Province production area is located in southwest China, just within this belt, and accounts for 98% of national coffee production. The remaining 2% mainly comes from Hainan. In fact, Pu’er city in Yunnan Province has been a prominent coffee grower in recent years, accounting for 95% of China’s coffee production (Zaman & Shan, 2024). *C. arabica* L. is the most important commercial species of coffee and is known for its unique taste, aroma, and health benefits; it can reduce inflammation in the body by influencing metabolic processes as well as prevent cardiovascular diseases (Chieng & Kistler, 2022; Surma et al., 2023).

Anthocyanins and carotenoids are key pigments in *C. arabica*, and their natural antioxidant properties have multiple health benefits (Khoo et al., 2017; Lozada-Ramírez et al., 2023). However, anthocyanins and carotenoids vary depending on the varieties of Costa Rican coffee (Esquivel et al., 2020). One previous study showed that anthocyanins were present in the red-colored fruit peels of coffee fruits, but they were low in the orange-colored fruit peels and not present in the yellow-colored fruit of coffee (Esquivel et al., 2020). Carotenoids affect the color of yellow-skinned varieties of coffee, and the levels of carotenoids and anthocyanins affect the color of red and orange coffee fruits (Esquivel et al., 2020).

The key genes related to anthocyanins and carotenoids are cinnamate 4-hydroxylase (C4H), phenylalanine ammonia lyase (PAL), chalcone synthase (CHS), flavonoid 3′-hydroxylase (F3′H), flavanone 3-hydroxylase (F3H), dihydroflavonol 4-reductase (DFR), anthocyanidin synthase (ANS), geranylgeranyl diphosphate (GGPS), phytoene desaturase (PDS), phytoene synthase (PSY), ζ-carotene desaturase (ZDS), carotene cis-trans isomerase (CRTISO), ζ-lycopene cyclase (LCYE), β-lycopene cyclase (LCYB), zeaxanthin epoxidase (ZEB), and others (Jaakola, 2013; Shen et al., 2019; Hu et al., 2022; Singh et al., 2024).

The main pigment compounds found in coffee, such as anthocyanins, proanthocyanidins and carotenoids are bioactive compounds found in coffee, which determine the color of coffee fruits and also have health benefits. The biochemical composition of coffee fruits varies according to genotype, and variations in the proportion and content of pigment compounds directly impact quality, taste, and commodity value (Tessema et al., 2011; Esquivel et al., 2020). At present, there are few multiomics studies on the difference of color metabolites and their expression regulation of coffee fruit. Therefore, this study sought to unravel the mechanisms of color formation in coffee by measuring metabolites and analyzing the expression of key metabolic pathways and key genes in three *C. arabica* fruits with substantial color differences.

## Materials and Methods

### Plant materials and sampling

**Figure 1:**
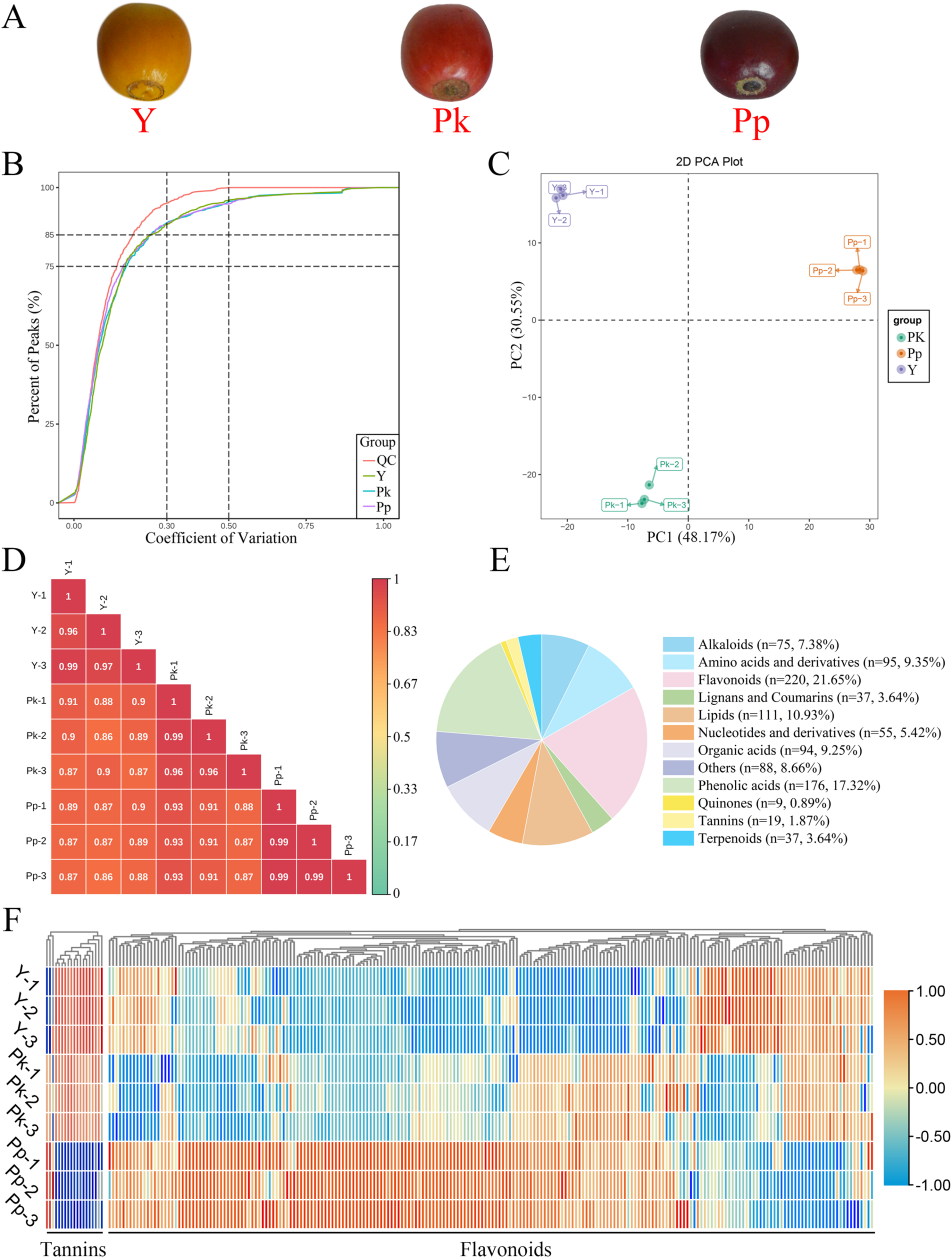
Phenotypes comparison and metabolome profiling of three coffee varieties. (A) Phenotypes of three coffee varieties. (B) CV value distribution diagram. The proportion of QC samples with CV value less than 0.5 exceeded 85%, indicating that the experimental data were stable. (C) PCA among biological replicates. (D) Intra-group and intergroup correlation of nine samples. (E) Classification and statistical analysis of all metabolites detected. (F) Heatmap of all flavonoid and tannin metabolites relative content. Red and green represent high and low metabolites content, respectively.

This study investigated three varieties of *C*. *arabica* belonging to the commercially important Catimor group, which is widely cultivated for its disease resistance and adaptability. The experimental materials were specifically selected based on their divergent fruit coloration at maturity—a key phenotypic trait linked to biochemical and potentially genetic differences. The varieties comprised DTAR1370 (yellow fruit, ‘Y’), DTAR1366 (pink fruit, ‘Pk’), and CatimorP4 (purple fruit, ‘Pp’). All samples were sourced from the Yunnan Provincial Seedling Breeding Base in Pu’er City, China (22°67′N, 100°88′E; altitude: 1,000–1,100 m) to ensure a uniform growing environment, with consistent field management protocols applied to all plants. To further standardize sampling, fully mature, pest- and disease-free fruits were exclusively harvested from the southeastern exposure of the coffee plant crown (Fig. 1A). Three independent biological replicates were performed for each coffee variety, and each biological replicate was a mixture of five to seven fruits. The harvested coffee fruits were snap-frozen in liquid nitrogen, then stored in a −80 °C environment for transcriptomic and metabolomic analyses.

### Extraction identification and quantitative analysis of metabolites

Metabolites of three *C. arabica* varieties were extracted with three biological replicates per cultivar and analyzed using a Ultra Performance Liquid Chromatography-Electrospray Ionization Tandem Mass Spectrometry (UPLC-ESI-MS/MS) system, according to the methods of Zhang et al. (2023). The anthocyanin and carotenoid contents of three *C. arabica* varieties were detected by MetWare (http://www.metware.cn/) based on the AB Sciex QTRAP 6500 LC-MS/MS platform.

### Metabolomic comparison and analysis

The principal component analysis (PCA) was performed using the R (http://www.r-project.org/) package prcomp and set function parameters: Skel = Tru. Data was scaled for unit variance prior to unsupervised PCA. Metabolite content data were normalized, and the heatmap was drawn by the R software Complex Heatmap package. Correlation analysis between samples was performed using Pearson’s correlation coefficients in R software and presented as heatmaps (Wei et al., 2017). The UpSet plot for differentially accumulated metabolites (DAMs) and the heatmap of metabolite contents in the anthocyanin and carotenoid metabolic pathways were generated using the TBtools software (Chen et al., 2020). Metabolites with |log 2-Fold Change| ≥ 2 and |log 2-Fold Change| ≤ 0.05 and VIP ≥ 1 evaluated in the Orthogonal Partial Least Squares-Discriminant Analysis (OPLS-DA) were considered as differentially accumulated metabolites. All metabolomics data were uploaded to the China National Center for Bioinformation (OMIX010390, https://ngdc.cncb.ac.cn/omix/preview/ABn6oFsn).

### RNA extraction and RNA-seq

Total RNA of *C. arabica* samples were extracted from the fruits using the RNA extraction kit (Tiangen DP441, Beijing, China). RNA-seq reads of the samples were obtained with an Illumina HiSeq 2000. Sequencing was performed on an Illumina sequencing platform, and the paired-end read length was 150 base paires (bp). The clean reads were mapped to the *C. arabica* reference genome (https://www.ncbi.nlm.nih.gov/datasets/genome/GCF_036785885.1) using HISAT2 (Kim, Langmead & Salzberg, 2015; Tran et al., 2018). The transcript abundance of each gene was measured using the fragments per kilobase of million mapped (FPKM). Genes with a |log 2 (fold change)| ≥ 1 and apadj value < 0.05 were considered differentially expressed genes (DEGs). Enriched DEG functions were functionally annotated using Kyoto Encyclopedia of Genes and Genomes (KEGG) pathway analyses (Hiroyuki & Susumu, 2000). The UpSet plot for DEGs were prepared using the OriginPro 8.5 software (OriginLab, Inc., Northampton, MA, USA), and the heatmaps of gene expression in the anthocyanin biosynthesis pathway and carotenoid biosynthesis pathway were generated with the TBtools software (Chen et al., 2020). Gene expression correlates with metabolite content, and chord plotting was performed using the Metware Cloud (https://cloud.metware.cn; Hu et al., 2021).

The statistical power of this experimental design, calculated in RNASeqPower (https://doi.org/doi:10.18129/B9.bioc.RNASeqPower) was 0.83 for yellow-colored fruits (Y), 0.84 for pink-colored fruits (Pk), and 0.73 for purple-colored fruits (Pp). Three technical replicates were used to achieve the statistical power.

### Quantification of gene expression

The primers of key genes were designed in Primer v3.0 (Table S1). Sample data were obtained from three biological repetitions and three technical replicates. Changes in relative expression were calculated with the 2^−ΔΔCt^ method (Livak & Schmittgen, 2001), and the *CaActin* gene (*LOC113777800*) was used as an internal reference gene (Martins et al., 2017).

## Results

### Metabolic differences among the fruits of three coffee varieties

**Figure 2:**
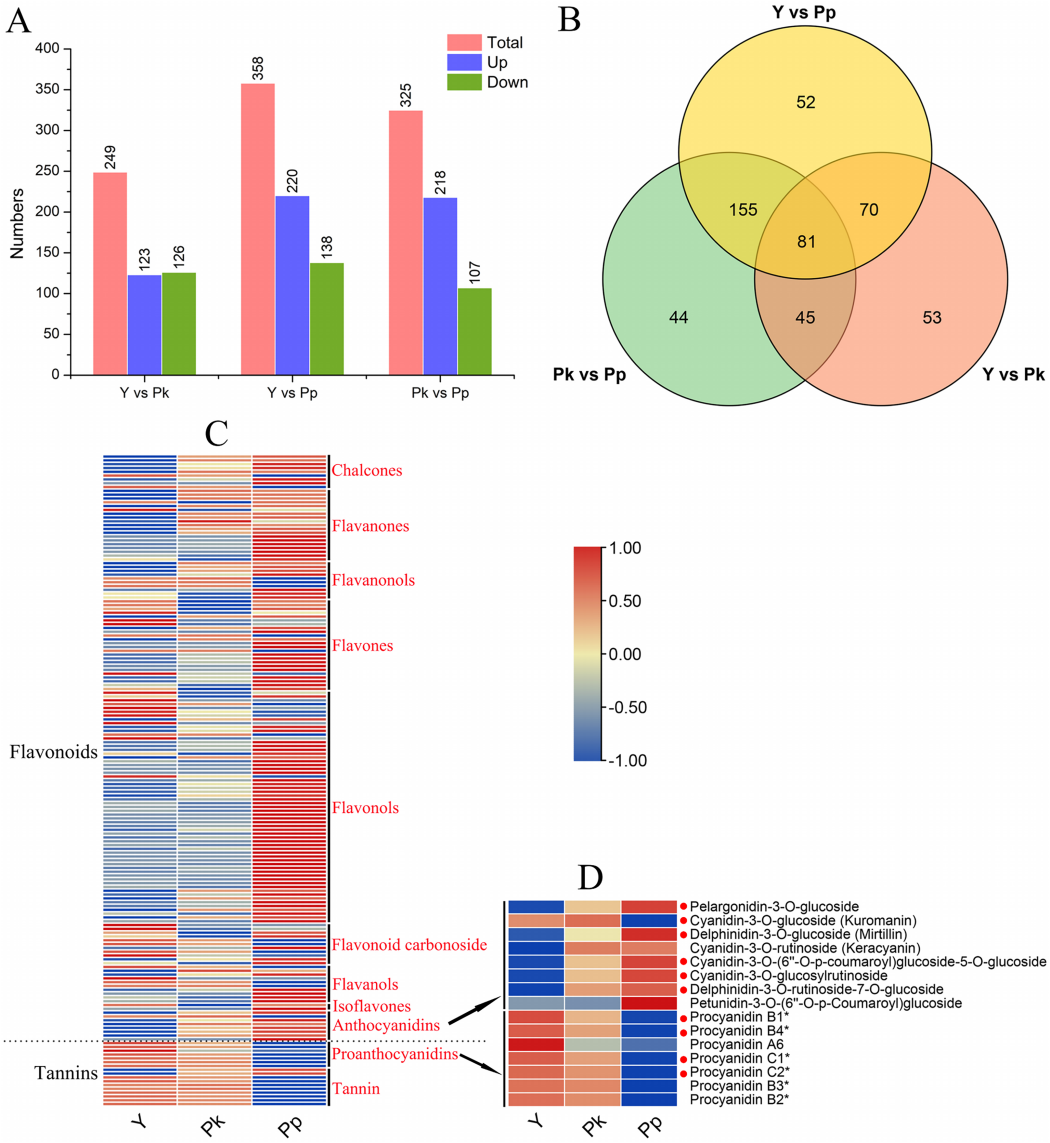
Analysis of differentially accumulated metabolites. (A) Comparison of up- and down-regulated DAMs among three coffee varieties. (B) Venn analysis of DAMs among Y *vs* Pp, Pk *vs* Pp, and Y *vs* Pk. (C) The heatmap analysis of DAMs in flavonoids and tannins. (D) The heatmap analysis of all differentially accumulated anthocyanins (DAAs) and differentially accumulated proanthocyanidins (DAPs) according to the relative content in samples. The metabolites highlighted with red dots represent the 81 common differential metabolites that belong to the three comparison groups in (B).

The differential flavonoids in nine samples of three coffee varieties were studied to comprehensively investigate metabolite differences, and a total of 1,016 metabolites were identified (Table S2). The metabolites with a Coefficient of Variation (CV) values less than 0.3 of QC samples in this study accounted for more than 85%, indicating the reliability of this experimental data (Fig. 1B). PC1 and PC2 accounted for 78.72% of the total variation, and there was a large separation between sample sets, indicating that there were significant differences in metabolites between the three *C. arabica* varieties (Fig. 1C). PC1 clearly distinguished the Pp treatment group from the other treatment groups, indicating that the metabolites in the Pp treatment group were more variable than those in the other treatment groups. Pearson’s correlation analysis demonstrated high correlation between repetitions, which further indicated the reliability of this experimental data (Fig. 1D). All metabolites of *C. arabica* were classified into 12 classes. The richest metabolites were flavonoids (220 metabolites), followed by phenolic acids (176 metabolites), lipids (111 metabolites), amino acids and derivatives (95 metabolites), and organic acids (94 metabolites), accounting for 21.65%, 17.32%, 10.93%, 9.35%, and 9.25% of total metabolites, respectively (Fig. 1E). Flavonoids had the most significant enrichment, and the relative content of 220 flavonoids was analyzed (Fig. 1F). As shown in Fig. 2F, the cluster analysis of metabolites in three coffee varieties had a clear accumulation pattern, indicating a high diversity of metabolites among the three different colored coffee varieties. Most of the flavonoids also showed a higher accumulation in the Pp group and a lower accumulation in the Y group, while the tannins, including proanthocyanidins, showed an opposite accumulation pattern.

### Metabolic differences among the fruits of three coffee varieties

Through the setting of thresholds for DAMs, the DAMs of three *C. arabica* fruits were compared (Fig. 2 and Table S3). The group Y *vs* Pp produced the highest number of DAMs (*n* = 358; 220 up-regulated and 138 down-regulated), followed by Pk *vs* Pp (*n* = 325; 218 up-regulated and 107 down-regulated), and the Y *vs* Pk group had the lowest number of DAMs (*n* = 249; 123 up-regulated and 126 down-regulated; Fig. 2A). Therefore, there were significant differences in the pattern of metabolite accumulation among the three coffee varieties. The number of unique DAMs in Y *vs* Pp, Pk *vs* pp, and Y *vs* Pk were 52, 44, and 53, respectively, with 112 overlapping DAMs in the combinations of Y *vs* PP and Pk *vs* Pp. A total of 81 DAMs were found in the three comparison groups (Fig. 2B). To further analyze the composition of DAMs in flavonoids and tannins, 171 DAMs were classified into eleven classes. Flavonols accounted for the highest proportion of flavonoids (61), followed by flavones (24) and flavanones (19; Fig. 2C). Eight DAMs of anthocyanidins (pelargonidin-3-O-glucoside, cyanidin-3-O-glucoside (Kuromanin), delphinidin-3-O-glucoside (Mirtillin), cyanidin-3-O-rutinoside (Keracyanin), cyanidin-3-O-(6″-O-p-coumaroyl)glucoside-5-O-glucoside, cyanidin-3-O-glucosylrutinoside, delphinidin-3-O-rutinoside-7-O-glucoside, and petunidin-3-O-(6″-O-p-Coumaroyl)glucoside) and seven DAMs of proanthocyanidins (procyanidins B1, B4, A6, C1, C2, B3, and B2) were identified in the three control groups, and their related contents were analyzed by heat map (Fig. 2D). The relative contents of the seven proanthocyanidins in the three coffee varieties were Pk > Y > Pp. Six of the eight DAMs of anthocyanidins (all but cyanidin-3-O-glucoside and petunidin-3-O-(6″-O-p-Coumaroyl) glucoside) showed an opposite pattern of relative content accumulation to proanthocyanidins. As seen in Fig. 2D, among the 15 anthocyanidins and proanthocyanidins, 10 compounds are DAMs shared by the three comparison groups in the Venn diagram analysis, which again indicates that anthocyanidins and proanthocyanidins have a significant regulatory effect on the color transformation of coffee fruits.

### Metabolomic analysis and quantitation of anthocyanin metabolites

**Figure 3:**
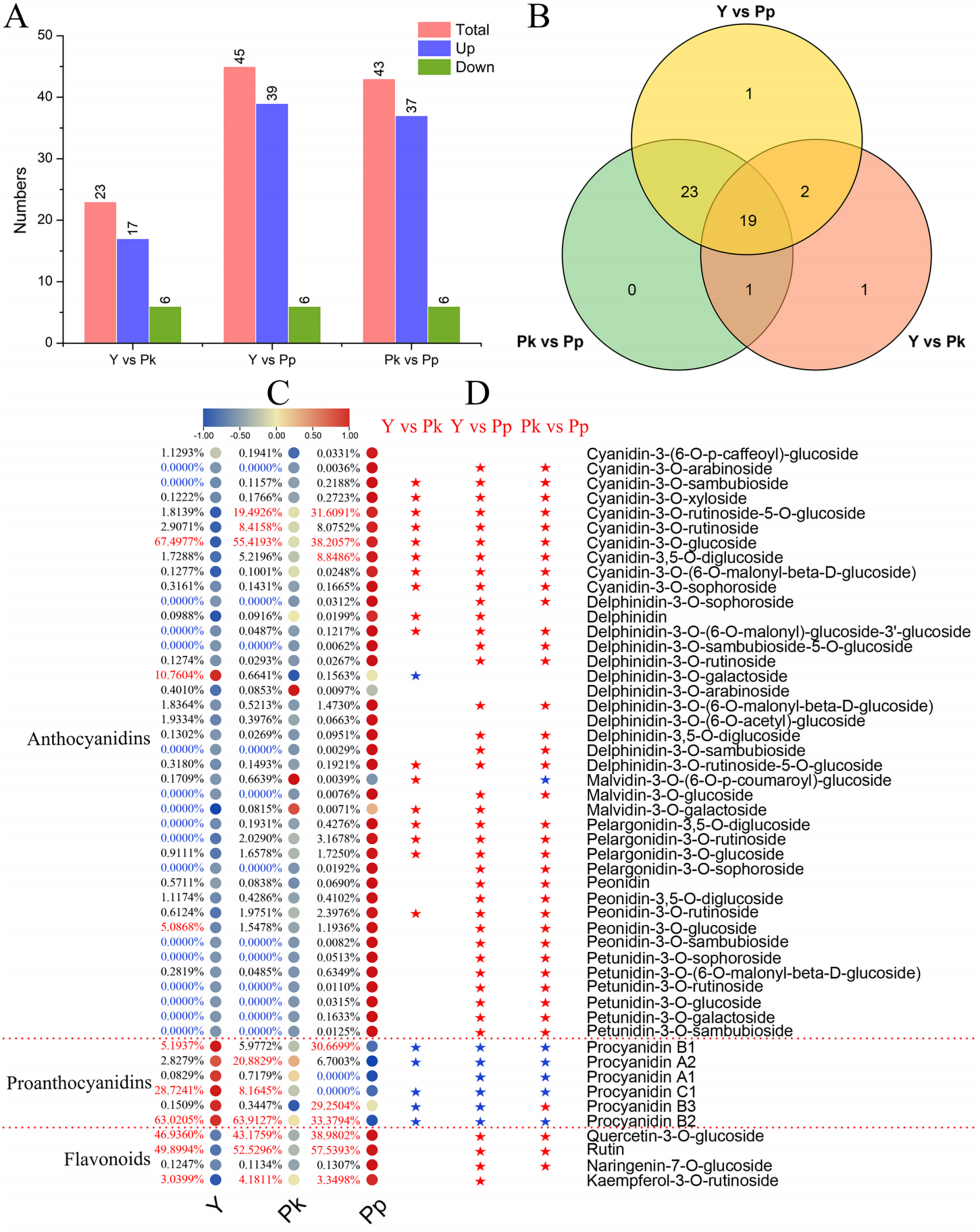
Differential accumulation and distribution of anthocyanins and proanthocyanidins in three different colors of coffee varieties. (A) Comparison of up- and down-regulated DAMs among three coffee varieties. (B) Venn analysis of DAMs among Y *vs* Pp, Pk *vs* Pp, and Y *vs* Pk. (C) Analysis of the difference in the proportion and relative content of anthocyanins and proanthocyanidins compounds. Values in blue are for compounds that were not detected in the sample, and the values in red are the top 3 compounds. (D) Significant analysis of metabolite differences in different comparison groups. The red and blue asterisks represent the up-regulated and down-regulated accumulation changes of the relative content of metabolites, respectively.

To fully understand the biochemical basis of *C. arabica* pigmentation, metabolite quantitation was conducted using a targeted database that contained 108 anthocyanins. Ultimately, 50 metabolites were identified by MS/MS in nine samples of three coffee varieties, including 40 anthocyanins (10 cyanidins, 12 delphinidins, four pelargonidins, three malvidins, five peonidins, and six petunidins), six proanthocyanidins, and four other flavonoids (Fig. 3C and Table S4). A targeted metabolome analysis showed that the Y *vs* Pk group had the lowest number of DAMs (23; 17 up-regulated and six down-regulated), and the Y *vs* Pp and Pk *vs* Pp groups produced similar DAMs (45 and 43; Fig. 3A, Table S5). Figure 3A shows that the number of up-regulated DAMs in the Pp group was significantly higher than down-regulated DAMs, indicating that the color mechanism of purple-colored fruits was more changed by the up-regulation and accumulation of metabolites compared with that of yellow and pink colored fruits. The Venn diagram analysis found that 19 overlapping differentially accumulated anthocyanins (DAAs) were shared among Y *vs* Pk, Y *vs* Pp, and Pk *vs* Pp (Fig. 3B), namely, cyanidin-3-O-sophorosid, pelargonidin-3,5-O-diglucoside, cyanidin-3-O-xylosid, cyanidin-3-O-rutinoside-5-O-glucoside, cyanidin-3-O-sambubioside, cyanidin-3-O-glucoside, cyanidin-3-O-rutinoside, delphinidin-3-O-rutinoside-5-O-glucoside, cyanidin-3,5-O-diglucosid, cyanidin-3-O-(6-O-malonyl-beta-D-glucoside), pelargonidin-3-O-glucoside, pelargonidin-3-O-rutinoside, procyanidin B2, procyanidin B1, procyanidin C1, procyanidin B3, delphinidin-3-O-(6-O-malonyl)-glucoside-3′-glucoside, peonidin-3-O-rutinoside, and procyanidin A2, which might be the key metabolites affecting fruit coloration in *C. arabica*.

To identify the correlation of DAMs with anthocyanin deposition in coffee fruit, the proportion of compounds in each sample and the relative content trends of comparisons of Y *vs* Pk, Y *vs* Pp, and Pk *vs* Pp were analyzed (Figs. 3C and 3D). Cluster analysis revealed different patterns of accumulation of anthocyanins, proanthocyanidins, and flavonoids (Fig. 3C). In the PP group, all 40 anthocyanins were detected, while only 33 and 38 compounds were detected in the Y group and Pk group, respectively. The following 12 compounds that were absent in the Pk group were also absent in the Y group: cyanidin-3-O-arabinoside, delphinidin-3-O-sophoroside, delphinidin-3-O-sambubioside-5-O-glucoside, delphinidin-3-O-sambubioside, malvidin-3-O-glucoside, pelargonidin-3-O-sophoroside, peonidin-3-O-sambubioside, petunidin-3-O-sophoroside, petunidin-3-O-rutinoside, petunidin-3-O-glucoside, petunidin-3-O-galactoside, and petunidin-3-O-sambubioside. Petunidins was the most absent compound, indicating that these co-absent compounds played an essential role in the purple coloring of *C. arabica* fruits. The five compounds that were specifically absent from yellow coloring of *C. arabica* fruits were cyanidin-3-O-sambubioside, delphinidin-3-O-(6-O-malonyl)-glucoside-3′-glucoside, malvidin-3-O-galactoside, pelargonidin-3,5-O-diglucoside, and pelargonidin-3-O-rutinoside. The top three compounds in yellow-colored *C. arabica* fruits were cyanidin-3-O-glucoside (67.4977%), delphinidin-3-O-galactoside (10.7604%), and peonidin-3-O-glucoside (5.0868%). The top three compounds in pink-colored *C. arabica* fruits were cyanidin-3-O-glucoside (55.4193%), cyanidin-3-O-rutinoside-5-O-glucoside (31.6091%), and cyanidin-3-O-rutinoside (8.4158%). The top three compounds in purple-colored *C. arabica* fruits were cyanidin-3-O-glucoside (38.2057%), cyanidin-3-O-rutinoside-5-O-glucoside (31.6091%), and cyanidin-3,5-O-diglucoside (8.8486%). These results show that the top three compounds with the largest proportion in red and purple *C. arabica* fruits were cyanidins. Therefore, cyanidins were considered to be major contributors to red pigmentation in *C. arabica* fruits. Cyanidin-3-O-glucoside is a major compound found at high levels in all fruits, and the cyanidin-3-O-glucoside content and accumulation of total anthocyanins both significantly declined from purple to pink and then from pink to yellow in *C. arabica* (Table S6). The cumulative content of anthocyanins in purple, pink, and yellow *C. arabica* fruits was 303.71, 36.79, and 7.51 μg/g, respectively.

In comparisons of Y *vs* Pk and Y *vs* Pp, the content of DAAs in pink and purple-colored *C. arabica* fruits was up-regulated and accumulated compared with the content of DAAs in yellow *C. arabica* fruits, with delphinidin-3-O-galactoside as an exception (Fig. 3D). Of the six proanthocyanidins identified in *C. arabica* fruits in this study, procyanidin A1 and procyanidin C1 were absent in purple *C. arabica* fruits (Table S7). The accumulation of proanthocyanidins showed an opposite trend to the accumulation of anthocyanins, and the cumulative content of proanthocyanidins in Y, Pk, and Pp was 1,037.17, 61.05, and 2.46 μg/g, respectively (Table S6). Among the 46 proanthocyanidins and anthocyanidins in this study, the top two compounds with the highest proportions in yellow, pink, and purple *C. arabica* fruits were, respectively, procyanidin B2 (653.63 μg/g) and procyanidin C1 (297.92 μg/g), procyanidin B2 (39.02 μg/g) and cyanidin-3-O-glucoside (20.39 μg/g), and cyanidin-3-O-glucoside (116.03 μg/g) and cyanidin-3-O-rutinoside-5-O-glucoside (96.00 μg/g). The content of proanthocyanidins and anthocyanidins procyanidin B2 and procyanidin C1, cyanidin-3-O-glucoside, cyanidin-3-O-rutinoside-5-O-glucoside and their derivatives enhanced the yellow and purple pigment deposition in *C. arabica* fruits. These results show that there were significant differences in anthocyanin and proanthocyanidin accumulations between Y and Pk, Y and Pp, and Pk and Pp.

### Metabolic analysis and quantitation of carotenoid metabolites

**Figure 4:**
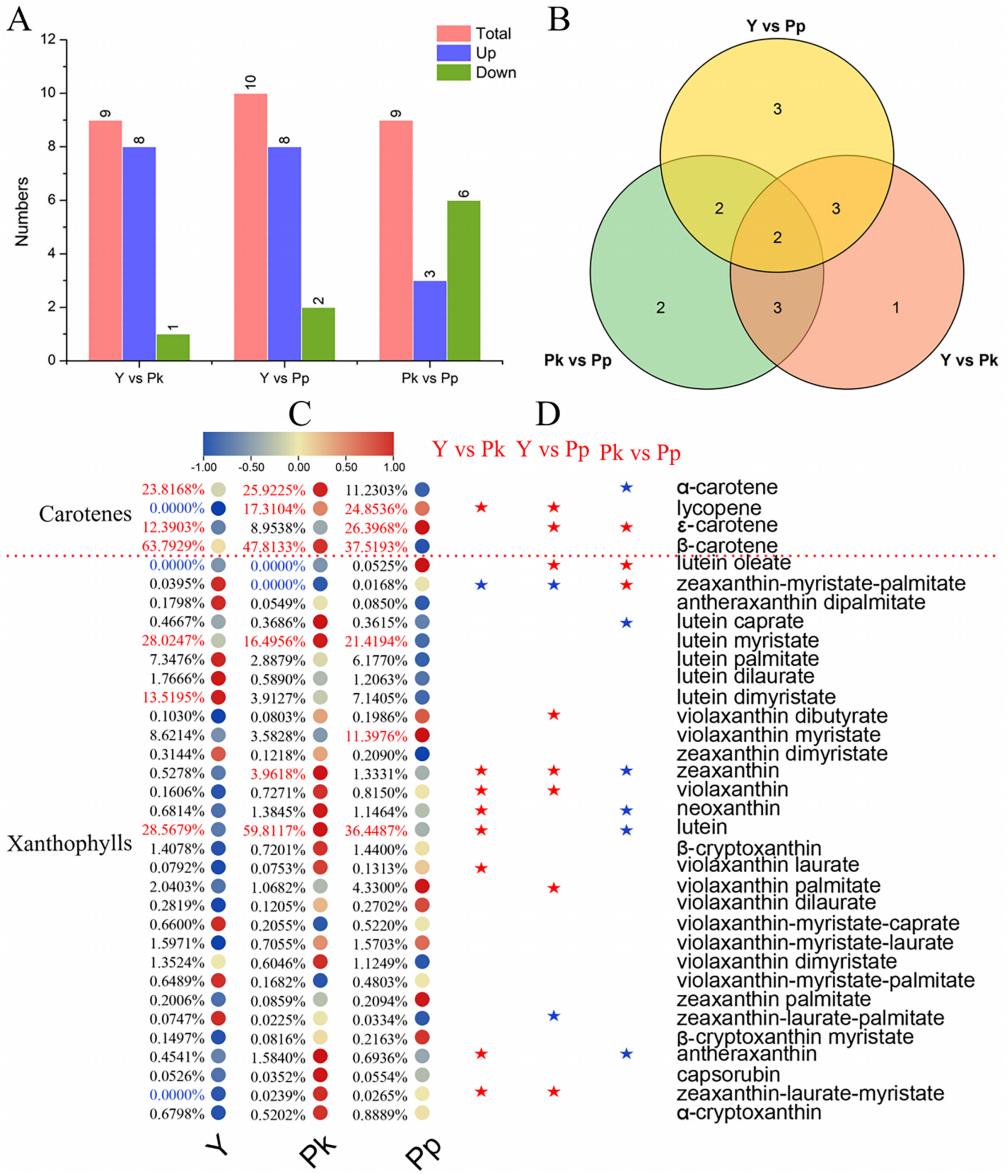
Differential accumulation and distribution of carotenoids in three different colors of coffee varieties. (A) Comparison of up- and down-regulated DAMs among three coffee varieties. (B) Venn analysis of DAMs among Y *vs* Pp, Pk *vs* Pp, and Y *vs* Pk. (C) Analysis of the difference in the proportion and relative content of carotenes and xanthophylls compounds. Values in blue are for compounds that were not detected in the sample, and the values in red are the top 3 compounds. (D) Significant analysis of metabolite differences in different comparison groups. The red and blue asterisks represent the up-regulated and down-regulated accumulation changes of the relative content of metabolites, respectively.

A total of 34 metabolites were identified by MS/MS in nine samples of three coffee varieties, including four carotenes and 30 xanthophylls (Fig. 4C and Table S7). The Y *vs* Pk, Y *vs* Pp, and Pk *vs* Pp groups all had similar Differentially Accumulated Carotenes (DACs) (nine, 10, and nine metabolites, respectively; Fig. 4A, Table S8). Compared with the Y group, the Pk and Pp groups showed more up-regulated changes and a higher accumulation of DACs. Venn diagram analysis showed that two overlapping DACs were shared among Y *vs* Pk, Y *vs* Pp, and Pk *vs* Pp (Fig. 4B), namely, zeaxanthin-myristate-palmitate and zeaxanthin. The Y *vs* Pp and Y *vs* Pk comparators also included three other common DACs, namely zeaxanthin-laurate-myristate, lycopene, and violaxanthin. These DACs may be the key metabolites influencing fruit coloration in *C. arabica*. To identify DAMs correlated with carotenoid deposition in coffee fruit, the proportions of compounds in each sample were analyzed as well as the relative content trends of the different comparison groups (Figs. 4C and 4D), and cluster analysis revealed different patterns of carotenes and xanthophylls (Fig. 4C). Lycopene, lutein oleate, and zeaxanthin-laurate-myristate were absent in the yellow-colored *C. arabica* fruits of this study, and lutein oleate and zeaxanthin-myristate-palmitate were absent in the Pk group. Lutein and lutein myristate significantly accumulated in *C. arabica* fruits, with the mean content of lutein in Pk, Pp, and Y being 64.58, 17.83, and 12.67 μg/g, respectively, and the mean content of lutein myristate in Pk, Pp, and Y being 17.81, 10.48, and 12.43 μg/g, respectively. Lutein and lutein myristate are the main contributors to the formation of carotenoids in *C. arabica* fruits. As the compound with the highest content of carotenoids, lutein and the accumulation of total carotenes and xanthophylls significantly decreased between pink and purple *C. arabica* fruits and then again between purple and yellow *C. arabica* fruits (Table S9). The cumulative content of carotenoids (including carotenes and xanthophylls) in pink, purple, and yellow *C. arabica* fruits was 115.11, 54.33, and 48.60 μg/g, respectively. In DAC comparisons of Y *vs* Pk and Y *vs* Pp, the content of DACs in pink and purple *C. arabica* fruits was up-regulated and significantly accumulated compared to yellow *C. arabica* fruits, with zeaxanthin-myristate-palmitate and zeaxanthin-laurate-palmitate being exceptions (Fig. 4D). Compared with the Pk group, six of the nine DACs down-regulated the accumulation change (including α-carotene, lutein caprate, zeaxanthin, neoxanthin, lutein, and antheraxanthin) in PP, and three up-regulated the accumulation change (ε-carotene, lutein oleate, and zeaxanthin-myristate-palmitate).

### Comparative transcriptome analysis

In this study, a total of 45,317,328–49,857,426 raw reads were generated from nine libraries in *C. arabica* fruits (Table S10). After filtration, a total of 42,267,334–45,636,644 clean reads were obtained, and the average Q20 and Q30 base rates were 97.53% and 93.00%, respectively, showing the overall sequence data was of high quality and suitable for further analysis.

**Figure 5:**
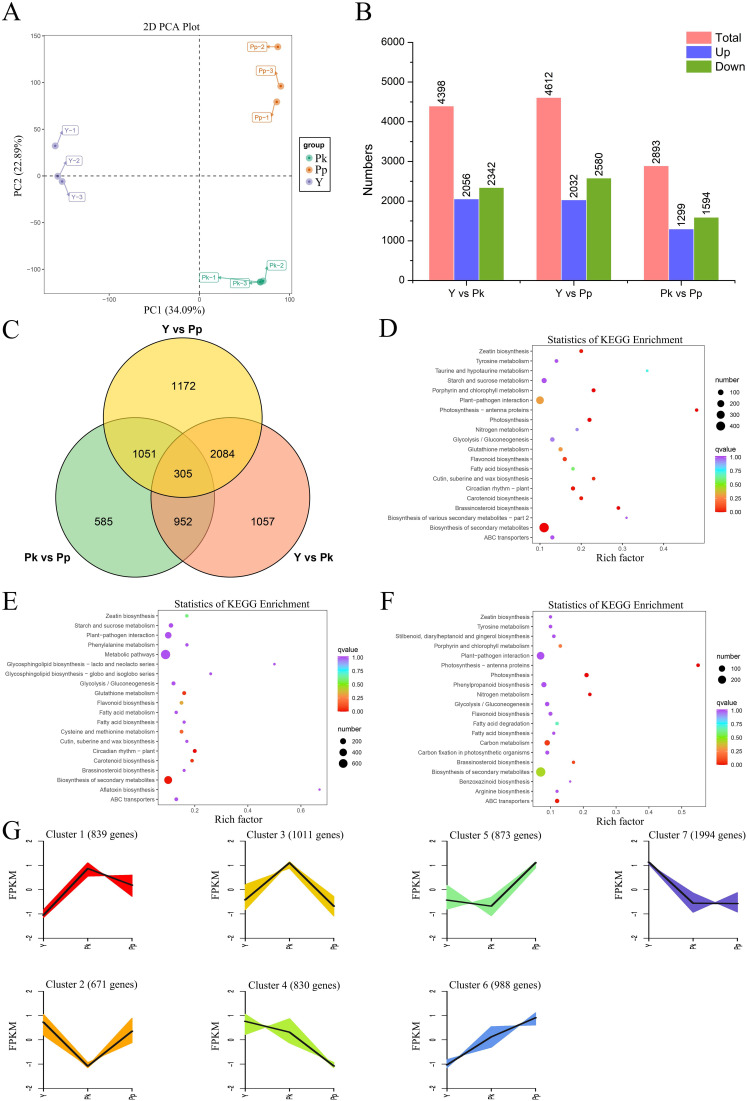
Gene regulation during *C. arabica* fruit color change. (A) PCA among biological replicates. (B) Up-regulation and down-regulation of DEGs. (C) Venn analysis among Y *vs* Pk, Y *vs* Pp, and Pk *vs* Pp. (D–F) Top 20 KEGG pathways with the most significant enrichment in the Y *vs* Pk, Y *vs* Pp and Pk *vs* Pp. (G) K-means cluster analysis of co-expression genes and their expression patterns.

The PCA analysis showed that the patterns of gene expression were highly consistent between different biological replicates, and the Y, Pk, and Pp groups were obviously divided on the PC1 × PC2 score plots, which indicated the high reliability of the sequencing data (Fig. 5A). The distribution of DEGs of *C. arabica* fruits in different treatment groups is shown in Fig. 5B. This study compared the up and down-regulation of DEGs in three sample pairs (Y *vs* Pk, Y *vs* Pp, and Pk *vs* Pp). A total of 5,066 (2,371 up-regulated, 2,695 down-regulated), 5,196 (2,315 up-regulated, 2,881 down-regulated), and 3,147 (1,406 up-regulated, 1,741 down-regulated) DEGs were identified in the Y *vs* Pk, Y *vs* Pp, and Pk *vs* Pp evaluations, respectively (Table S11). The most significant number of DEGs were compared with the Y group (Y *vs* Pk and Y *vs* Pp), and more of the DEGs showed down-regulated changes than up-regulated. Further analysis identified 362 shared DEGs between the three comparison groups (Fig. 5C). KEGG pathway enrichment analysis of the DEGs showed the top 20 enriched pathways (Figs. 5D–5F). “Carotenoid biosynthesis” was significantly enriched in different treatment groups, suggesting that these differentially expressed genes were highly related to carotenoid cumulation in *C. arabica* fruits. In addition, “Flavonoid biosynthesis” was significantly enriched compared with group Y (Y *vs* Pk and Y *vs* Pp). A K-means cluster analysis was performed using the FPKM values of the Y, Pk, and Pp transcriptome data in *C. arabica* fruits to infer potential transcriptional regulatory relationships and identify co-expressed genes in the DEGs of *C. arabica* fruits (Fig. 5G). During the color change of *C. arabica* fruits, three clusters were found, and each cluster had similar molecular functions. Among these clusters, the expression levels of Cluster one genes in Y and Pp were similar and low, while these genes were significantly up-regulated in the Pk group. The expression levels of Cluster three genes in Y and Pk were low, but were significantly up-regulated in Pp. The expression levels of Cluster two genes in Pk and Pp were similar and low, but were significantly up-regulated in Y.

### Phenylpropanoid, flavonoid, and anthocyanidin biosynthesis pathway during *C. arabica* fruit color change

**Figure 6:**
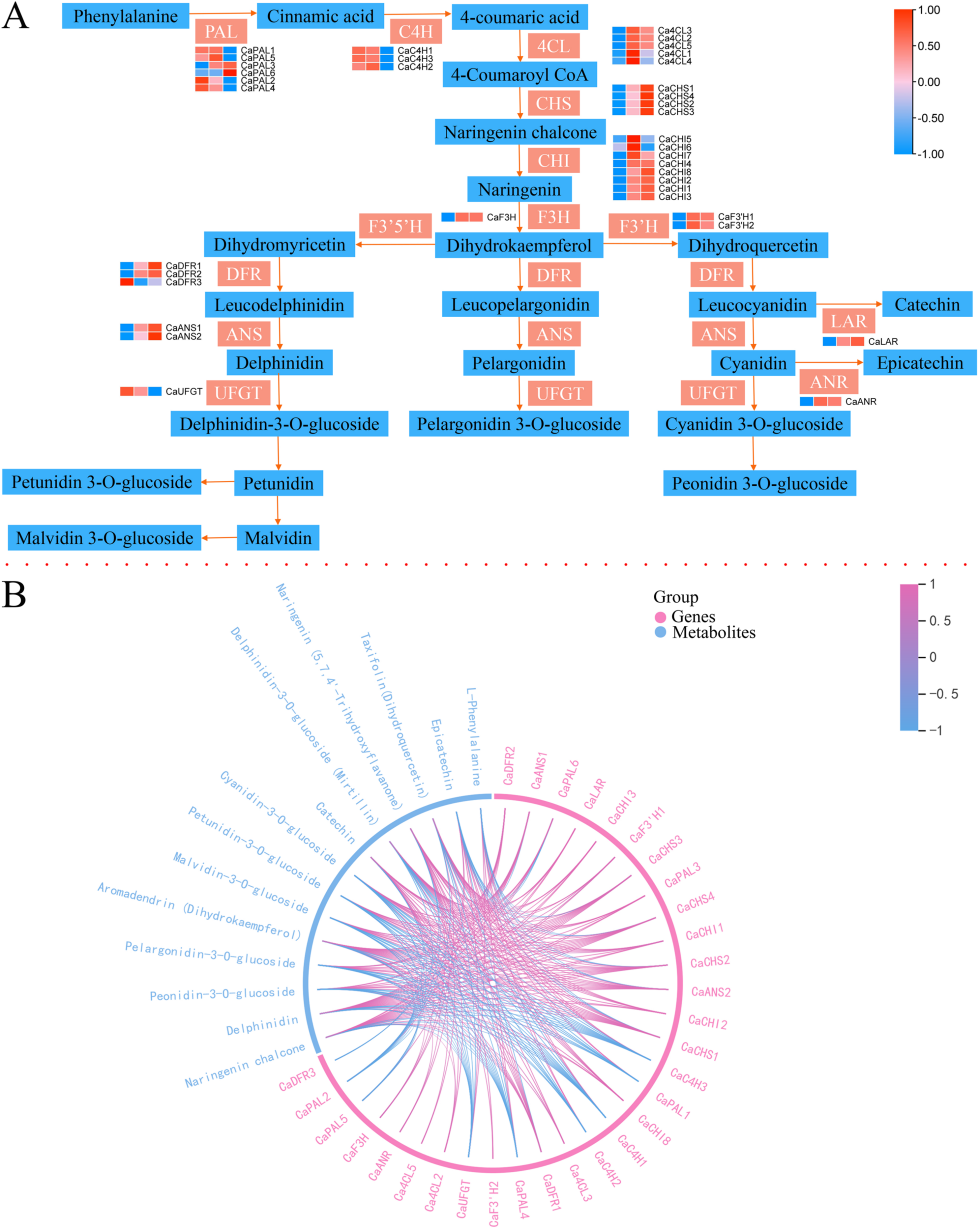
Expression of structural genes in the anthocyanin biosynthesis pathway and connection network between the key DEGs and DAAs. (A) Reconstruction of the anthocyanins biosynthetic pathway with the structural genes. (B) Connection network between the key DEGs and DAAs. The blue markings on the outside of the circle represent DAAs, and the red markings on the outside of the circle represent DEGs; The red line in the circle indicates a positive correlation at the 0.05 level, and the blue line s indicates a negative correlation at the 0.05.

To understand the key genes of anthocyanins based on phenotypic data, the transcriptome and metabolome data of anthocyanin biosynthesis of three different colors of *C. arabica* were integrated, and the complete metabolic pathway of anthocyanin biosynthesis was reconstructed; the genes with a mean FPKM of less than five in the three coffee samples were removed from the pathway (Fig. 6A, Table S12). The transcript abundance of eight key genes (four *CaPALs*, two *CaC4Hs*, and two *CaUFGTs*) was lower in Pp than in Y and Pk. The expression level of *CaCHSs, CaCHIs, CaF3Hs, CaANSs, and CaLARs* was the highest in the Pp group, followed by the Pk group, and was the lowest in the Y group. *CaF3′Hs* was the lowest in group Y. Correlation and chord diagrams of key metabolites and genes in the anthocyanin biosynthesis pathway were plotted (Fig. 6B). The content of delphinin and naringenin chalcone metabolites presented significant positive correlations with the expression levels of *CaANSs*, *CaCHSs*, *CaDFRs*, *CaPAL3*, *CaPAL4*, *CaLARs*, *CaCHIs*, and *CaF3Hs*, and significant negative correlations with the expression levels of *CaC4H1*, *CaPAL2*, *CaPAL8, CaPAL11*, and *CaUFGTs*. Naringenin chalcone was significantly positively correlated with *Ca4CL2, Ca4CL4, Ca4CL7*, and *CaF3′H1*. The content of petunidin-3-O-glucoside, cyanidin-3-O-glucoside, and malvidin-3-O-glucoside presented significant positive correlations with *CaANS1*, *CaANS2*, *CaF3H1* and *CaCHSs*, and negative correlations with *CaC4Hs*, *CaPAL1*, *CaPAL2*, *CaPAL8*, *CaPAL11, CaANR*, and *CaUFGT*. Cyanidin-3-O-glucoside also had a significant positive correlation with *CaDFRs*, *CaF3H2*, *CaLAR1*, *CaPAL3*, and *CaCHI1*. Peonidin-3-O-glucoside and pelargonidin-3-O-glucoside levels presented significant positive correlations with *CaCHSs*, *CaDFR3*, *CaANS1, CaANS2*, and *CaF3H1*, and negative correlations with *CaC4Hs*, *CaPAL1*, *CaPAL2*, *CaPAL8*, *CaPAL11*, and *CaUFGT1*. Naringenin (5,7,4′-Trihydroxyflavanone) was significantly positively correlated with several key genes including *CaANS1*, *CaCHIs*, *CaCHS1*, *CaLARs*, *CaF3′Hs*, *Ca4CLs*, *CaDFRs*, and *CaF3Hs*.

### Carotenoid biosynthesis pathway during *C. arabica* fruit color change

**Figure 7:**
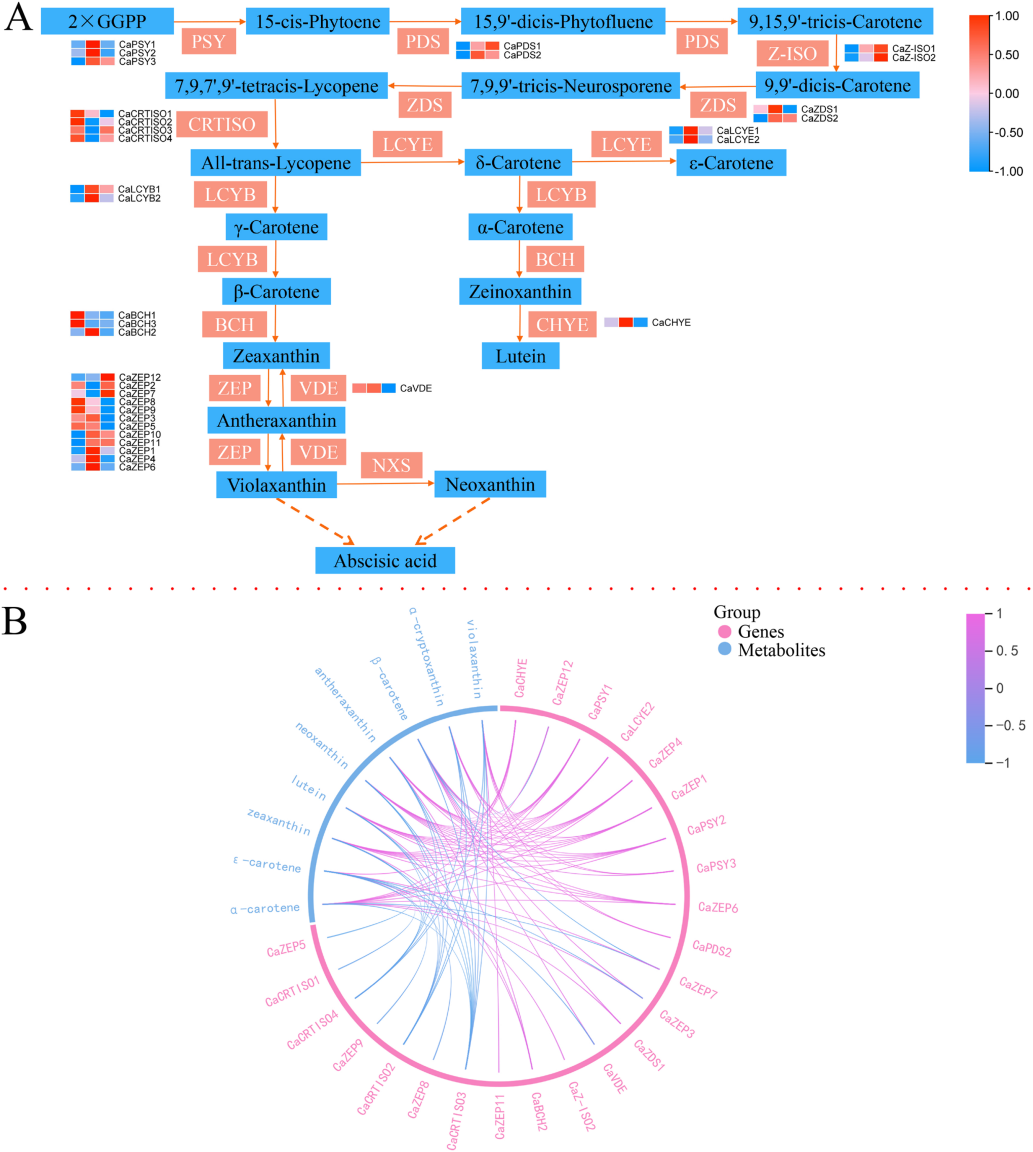
Expression of structural genes in the carotenoid biosynthesis pathway and connection network between the key DEGs and DACs. (A) Reconstruction of the anthocyanins biosynthetic pathway with the structural genes. (B) Connection network between the key DEGs and DACs. The blue markings on the outside of the circle represent DAAs, and the red markings on the outside of the circle represent DEGs; The red line in the circle indicates a positive correlation at the 0.05 level, and the blue lines indicates a negative correlation at the 0.05.

Transcript levels of 44 genes of *C. arabica* fruits related to carotenoid biosynthesis were analyzed using the RNA-seq data (Fig. 7A and Table S13). Among these genes, *CaPSYs*, *CaLCYE*, *CaCHYE3, CaCHYE5*, *CaVDEs*, *CaLCYBs*, and most *CaZEPs* were highly expressed in the Pk group. Key genes such as *CaPSYs*, *CaPDSs*, *CaZ-ISO*, *CaLCYE*, and *CaLCYBs* were lower in the Y group. The results of the correlation analysis showed that violaxanthin and α-cryptoxanthin contents presented significant positive correlations with *CaPSY1*, *CaPSY3*, *CaZDS2*, *CaZEP3*, *CaZEP11*, and *CaPDS2*. Violaxanthin, α-cryptoxanthin, antheraxanthin, neoxanthin, lutein, and zeaxanthin presented significant negative correlations with *CaCRTISO4*, *CaCRTISO5*, and *CaCRTISO6* (Fig. 7B). β-carotene presented significant positive correlations with *CaCHYE3*, *CaCHYE5, CaZEP8*, *CaZEP9*, *CaZEP13*, *CaPSY1*, *CaPSY2*, *CaZDS1, CaVDE2*, and *CaVDE3*, and significant negative correlations with CaZEP12, *CaCHYE4*, and *CaCRTISO5*. Antheraxanthin presented significant negative correlations with *CaCHYE3*, *CaCHYE5*, *CaPSY1*, *CaPSY2*, *CaZDS2*, *CaZEP9*, and *CaBCH2*. Antheraxanthin, zeaxanthin, and lutein presented significant positive correlations with *CaCHYE3*, *CaCHYE5*, *CaPSY1*, *CaPSY2*, *CaLCYE9*, and *CaZDS2*. Neoxanthin presented significant positive correlations with *CaCHYE5*, *CaPSYs*, *CaZDS2*, *CaZEP3*, *CaZEP9*, and *CaZEP11*. ε-carotene presented significant positive correlations with *CaZEP12*, *CaZEP2*, and *CaZ-ISO*, and significant negative correlations with *CaZEP8*, *CaVDEs*, *CaZEP10*, *CaZEP13*, *CaZEP15, CaZEP17, CaCRTISO2*, and *CaCRTISO1*. α-carotene presented significant positive correlations with *CaCHYE3, CaCHYE5*, *CaPSY1*, *CaPSY2*, *CaZEP8, CaZEP9, CaVDE2*, and *CaZDS1*, and significant negative correlations with *CaZEP12, CaCHYE4*, and *CaCRTISO5*.

### Validation of the expression levels of detected DEGs in three *C. arabica* fruits

**Figure 8:**
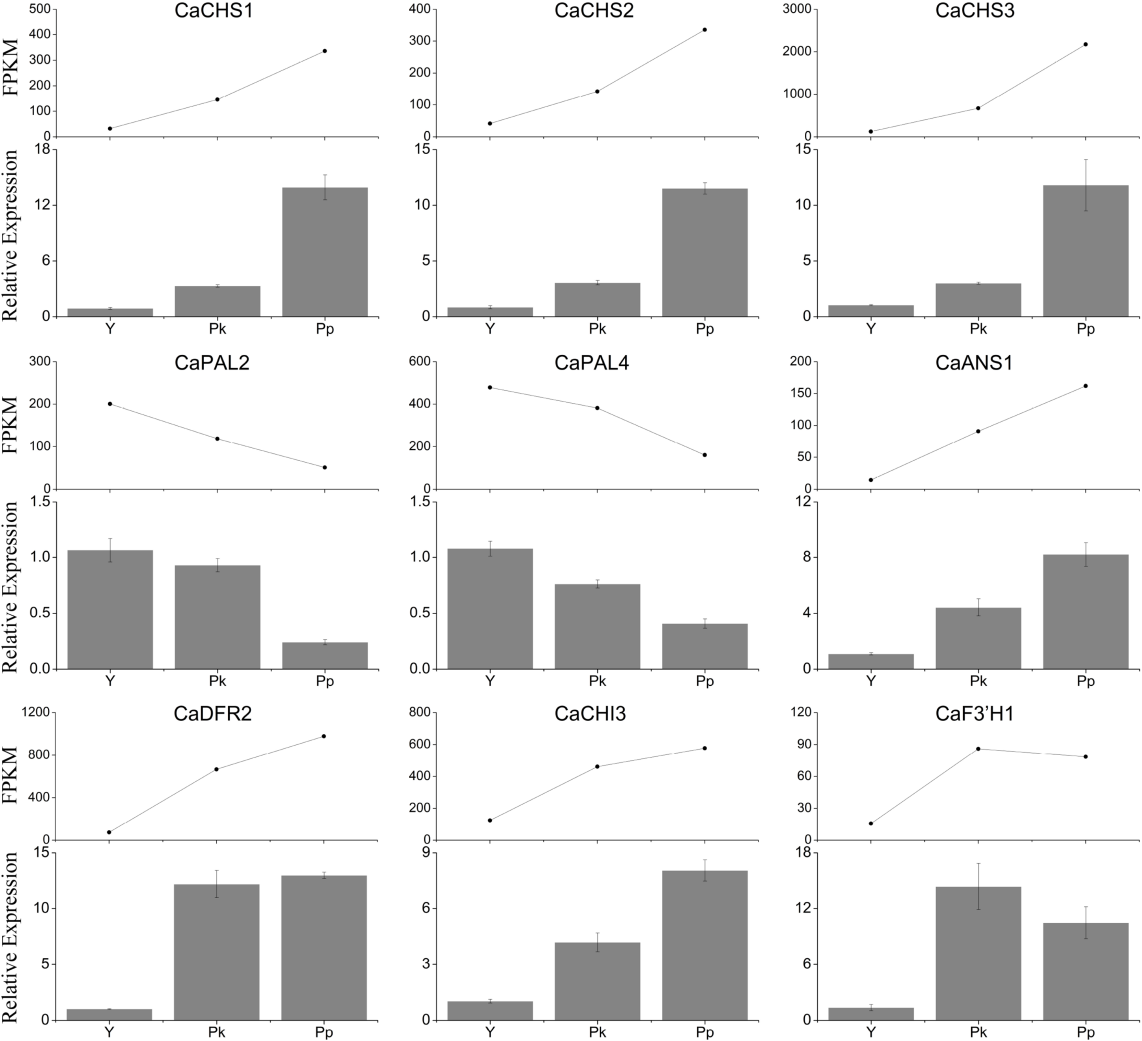
qRT-PCR validation of the gene’s expression levels of *C*. *arabica* fruits selected from the transcriptome data.

A qRT-PCR gene expression analysis was performed using the nine key genes in the anthocyanin metabolism pathway to verify the transcriptome data from the RNA-seq analysis of three *C. arabica* fruits (Fig. 8). The results of the RNA-seq and qRT-PCR analyses were consistent.

## Discussion

### Validation of the expression levels of detected DEGs in three *C. arabica* fruits

Anthocyanins, proanthocyanidins, and carotenoids contribute to diverse plant pigmentation and changes (Fu et al., 2021; Liu et al., 2024). Anthocyanins are characteristic compounds that contribute to the attractive color of fruits (Saigo et al., 2020), but the specific biochemicals that contribute to the metabolic differences in the color of coffee are not well understood. For this reason, metabolites were quantified for anthocyanins and carotenoids through the targeted database and based on an analysis of the flavonoids and anthocyanins data obtained from the metabolome. The biochemical basis of yellow, pink, and purple coloring of *C. arabica* fruits was systematically analyzed in this study. The contents of anthocyanins and carotenoids were significantly and differently enriched in the three different colors of *C. arabica* fruits. Anthocyanidins and proanthocyanidins have a vital effect on the color variation of *C. arabica* fruits. Analysis of the flavonoids and anthocyanins data obtained from the metabolome indicated that the metabolites in the purple coloring of *C. arabica* fruits were more variable than those in yellow and pink coloring of *C. arabica* fruits, and the color mechanism of purple-colored fruits was more distinguished by the up-regulation and accumulation of metabolites compared with that of the yellow and pink fruits (Figs. 2A and 3A). The up-regulation and accumulation of most flavonoids, including anthocyanidin compounds, and the down-regulation of proanthocyanidins were important factors for the purple pigmentation of *C. arabica* fruits, though the down-regulation of anthocyanins and the up-regulation accumulation of proanthocyanidins were important factors affecting the yellow pigmentation of *C. arabica* fruits. These results are somewhat similar to the observed anthocyanin deposition in tubers of potato that ultimately caused pink, red, and purple coloring (Oertel et al., 2017; Zhang et al., 2023). We also noted that the anthocyanins involved in the three *C. arabica* fruits were cyanidin derivatives, which accounted for 75.64%, 89.28%, and 87.46% of the total anthocyanin metabolites in Y, Pk, and Pp, respectively. These results are consistent with the results of a leaf study of Chokecherry (*Padus virginiana* L.), where the proportion of cyanidins was higher in purple and purple-red leaves (Li et al., 2021). In the present study, the top three compounds with the largest proportion of red and purple coloring of *C. arabica* fruits were cyanidins. Therefore, cyanidins were considered to be major contributors to redness in anthocyanins of *C. arabica* fruits (Esquivel et al., 2020). Cyanidin-3-O-glucoside content and accumulation of total anthocyanins showed a significant decrease from purple to pink and then from pink to yellow in *C. arabica*. These same patterns were observed in our previous studies on Chinese cherry [*Cerasus pseudocerasus* (L.) G.Don], in which the total anthocyanins diminished from black purple to red and then from red to yellow in Chinese cherry fruits (Liu et al., 2024). The anthocyanin content in the purple petals of *Scutellaria baicalensis* has also been found to be significantly higher than that in the pink and white petals (Hu et al., 2023).

Compounds such as cyanidin-3-O-rutinoside, delphinidin-3-O-glucoside, pelargonidin-3-O-glucoside, and peonidin-3,5-O-diglucoside were significantly up-regulated and accumulated in purple *C. arabica* fruits, similar to the observed results of purple pigmentation in potato tubers (Zhang et al., 2023). As the main metabolite of purple coloring of *C. arabica* fruits, anthocyanins had a much higher content level (303.71 μg/g) than that of proanthocyanidins (2.46 μg/g), and 83% of the petunidans were unique to the purple coloring of *C. arabica* fruits. Therefore, cyanidin derivatives and petunidan derivatives are considered to be important factors in the purple coloring of *C. arabica* fruits. This finding differs from the results of potato studies, which found that in purple potato tubers, malvidin, petunidin, and delphinidin were the most contributing metabolites, while in purple coloring of *C. arabica* fruits, malvidins contributed the least (only 0.06 μg/g; Zhang et al., 2023). The total content of procyanidins (61.05 in Pk and 1,037.17 in Y) in pink and yellow *C. arabica* fruits was significantly higher than that of total anthocyanidins (36.79 in Pk and 7.15 in Y). Among the proanthocyanidin derivatives, Procyanidin B2 was the highest in the three coffee fruits, and the contents of Procyanidin B2 (653.63 μg/g) and Procyanidin C1 (297.92 μg/g) in yellow *C. arabica* fruits accounted for 92% of the total content of proanthocyanidins, which was different from the Chokecherry study results where the expression levels of procyanidin B2 and proanthocyanidins C1 were the highest in green and purple leaves, respectively (Li et al., 2021). In the present study, cyanidins, delphinidins, pelargonidins, and peonidins maintained higher levels in Pk and Pp fruits compared to Y fruits, which was similar to the results of a quantitative exploration of the anthocyanin components of *S. baicalensis* petals, which showed that cyanidin, pelargonidin, and delphinidin in purple-F and pink-F *S. baicalensis* petals maintained reasonably high levels, which the authors speculated may have contributed to the formation of purple and pink pigments in the petals (Hu et al., 2023). Carotenoids help fruits and vegetables turn yellow or red. In tubers, about 80% of carotenoids are violetxanthin, lutein, or zeaxanthin; the lutein miristat in the yellow potato tuber was significantly higher than in the white tuber (Nesterenko & Sink, 2003; Zhang et al., 2023). In the present study, lutein and lutein myristate significantly accumulated in three *C. arabica* fruits, and they were considered to be major contributors to carotenoids in *C. arabica* fruits. As the compound with the highest content of carotenoids, lutein and the accumulation of total carotenes and xanthophylls significantly decreased from pink to purple and then from purple to yellow in *C. arabica* fruits. Lycopene is the main coloring pigment of the red color of fruits, which catalyzes to form zeaxanthin or lutein (Li et al., 2022). Previous studies on papaya (*Carica papaya* L.) have shown that lycopene is the main carotenoid in the flesh of red-fleshed papaya, and β-carotene and β-cryptoxanthin are the main carotenoids in the flesh of yellow-fleshed papaya (Shen et al., 2019). The red color of red pepper pods (*Capsicum annuum* L.) is also due to the presence of carotenoids, with zeaxanthin also contributing to the red color (Schweiggert et al., 2005). In the present study, three unique DACs were included in the Y *vs* Pp and Y *vs* Pk comparison groups, namely lycopene, zeaxanthin-laurate-myristate, and violaxanthin. Lycopene, lutein oleate, and zeaxanthin-laurate-myristate were absent in the yellow *C. arabica* fruits, indicating lycopene and zeaxanthin-laurate-myristate contribute to the red color of *C. arabica* fruits.

### Identification of key genes controlling anthocyanin and carotenoid biosynthesis and metabolic divergence

The anthocyanin biosynthesis process is relatively clear and conserved, and the key regulation structural genes mainly include PAL, C4H, 4CL, CHS, F3H, F3′H, F3′5′H, DFR, ANS, and UFGT (Jaakola, 2013). PAL, C4H, 4CL, CHS, CHI, F3H, and F3′H, as upstream key regulatory genes for anthocyanin synthesis, were roughly divided into two expression patterns in coffee fruits. Most of the *CaPAL* and *CaC4Hs* were low in the Pp *C. arabica* fruits, while *Ca4CL*, *CaCHS*, *CaCHI*, *CaF3H*, and *CaF3′H* were low in the Y *C. arabica* fruits. CHS catalyzes the production of chalcones, using CoA and 4-coumaroyl CoA as substrates. Chalcone synthesis compounds produce naringentin, an important substrate for flavonoid synthesis, under the action of CHI, and F3H forms dihydroflavonol through the catalysis of the substrate (Winkel-Shirley, 2001). CHS, CHI, and F3H constitute the key enzymes in flavonoid synthesis (Nishihara, Nakatsuka & Yamamura, 2005). In this study, naringenin chalcone and naringenin presented significant positive correlations with the expression levels of *CaCHS1*, *CaCHIs*, *CaF3Hs*, *Ca4CL2, Ca4CL4*, and *Ca4CL7*, and naringenin chalcone was also positively correlated with *CaCHS2*, *CaCHS3*, and *CaCHS4*, which showed low expression in Y and high expression in Pp. These results indicate that *CaCHSs*, *CaCHIs*, *Ca4CLs*, and *CaF3Hs* could help enhance the synthesis of important compounds in the upstream pathway of anthocyanin synthesis, including chalcone, naringenin, and dihydroflavonol. *F3′H* converted dihydrokaempferol into different pathways, producing red pigments (Liu et al., 2023). Two homologues of *CaF3′H* in *C. arabica* fruits were identified and were significantly up-regulated in Pk and significantly decreased in Y, consistent with the results of the study of potato tubers (Zhang et al., 2023), indicating that *CaF3′Hs* has a potential role in promoting metabolic influx into cyanidin and peonidin-related biosynthesis. Dihydroflavonols form leukanthocyanins under the action of DFR and then synthesize the corresponding-colored anthocyanins by ANS. Then, under the catalysis and modification of UFGT and AOMT, stable anthocyanins are finally formed (Zhang, Butelli & Martin, 2014; Zhao & Tao, 2015). The changes of metabolite content in delphinidin, cyanidin-3-O-glucoside, petunidin-3-O-glucoside, and malvidin-3-O-glucoside were significantly positively correlated with the expression of the *CaANSs* gene and negatively correlated with *CaUFGT1*, and the content of metabolites was the lowest in Y and the highest in Pp. Delphinidin and cyanidin-3-O-glucoside were significantly positively correlated with *CaDFRs*. These results suggest that *CaANSs* and *CaDFRs* were highly involved in anthocyanin deposition in *C. arabica* fruits, controlling the generation of purple- and red-related anthocyanin. Finally, the carotenoids biosynthetic pathway and the key genes in plants has been gradually clarified. Two GGPP molecules form lycopene *via* PSY, PDS, and ZDS (Giuliano, Aquilani & Dharmapuri, 2000; Hirschberg, 2001). Located upstream from GGPP to carotene in this study, expressions of PSY, PDS, Z-ISO, ZDS, and CRTISO were identified. The expressions of *CaPSYs*, *CaPDSs*, *CaZ-ISO*, and *CaZDS2* showed a low level in Y, which was consistent with the lycopene accumulation pattern in *C. arabica* fruits, and lycopene was not detected in yellow *C. arabica* fruits. Therefore, *CaPSYs*, *CaPDSs*, CaZ-*ISOs*, and *CaZDS2* were important to the formation of lycopene in *C. arabica* fruits. After the biosynthesis of lycopene into the α-branch and β-branch, the LCY catalyzes the cyclization of lycopene to form α-carotene and β-carotene, respectively. α-carotene is converted into lutein by BCH and CHYE; β-carotene, through BCH, ZEP, and VDE, contributes to zeaxanthin, antheraxanthin, and violaxanthin production (Giuliano, Aquilani & Dharmapuri, 2000; Hirschberg, 2001; Wolters et al., 2010). The *CaLCYE* and *CaLCYBs* showed the highest expression in Pk and a low level in Y. The analysis of correlation and chord diagrams showed that the content ofα-carotene and lutein had a significant positive correlation with the expression levels of *CaCHYE3* and *CaCHYE5*. *CaCRTISO4*, *CaCRTISO5*, and *CaCRTISO6* negatively regulated the biosynthesis of downstream carotenoids including violaxanthin, antherxanthin, neoxanthin, lutein, and zeaxanthin. *CaZEP9* had a significant positive correlation with the downstream carotenoid biosynthesis in the β-branch. These results indicated that the gene expression of key structural genes *CaPSYs*, *CaPDSs*, *CaZ-ISO*, *CaZDS2*, *CaCHYE3*, *CaCHYE5*, *CaZEP9*, and *CaCRTISOs* in *C. arabica* fruits contributed to carotenoid biosynthesis.

## Conclusions

We employed multiomics techniques in this investigation to conduct a systematic examination of the biochemical underpinnings of the yellow, pink, and purple colors of *C. arabica* fruits. The KEGG enrichment analyses demonstrated that the DEGs and DAMs were significantly involved in the metabolism of carotenoids and anthocyanins. Cyanidin and its derivatives comprised over half of the anthocyanins in the three *C. arabica* fruits. The accumulation of proanthocyanidins was responsible for the purple coloration. The primary cause of the red color in the anthocyanins of *C. arabica* fruits was believed to be cyanidin-3-O-glucoside compounds. In the pathway that precedes anthocyanin synthesis, the main target genes *CaCHSs*, *CaCHIs*, *Ca4CLs*, and *CaF3Hs* may serve to increase the production of these critical compounds. Anthocyanin deposition was facilitated by *CaANSs* and *CaDFRs*, while lycopene formation was facilitated by *CaPSYs*, *CaPDSs*, *CaZ-ISO*, and *CaZDS2*. *CaPSYs*, *CaPDSs*, *CaZ-ISO*, *CaZDS2*, *CaCHYE3*, *CaCHYE5*, *CaZEP9*, and *CaCRTISOs* were key genes that were regulated in *C. arabica* fruits to assist in the production of carotenoids. Consequently, this study offers additional insights into the regulation of *C. arabica* fruit color formation and the enhancement of fruit quality and appearance.

## Supplemental Information

10.7717/peerj.20305/supp-1 Supplemental Information 1 Specific primer pairs.

10.7717/peerj.20305/supp-2 Supplemental Information 2 Identification of metabolite in three coffee varieties.

10.7717/peerj.20305/supp-3 Supplemental Information 3 Identification of differentially accumulated metabolites (DAMs) in different groups.

10.7717/peerj.20305/supp-4 Supplemental Information 4 Identification of anthocyanins contents (μg/g) in coffee samples based on LC-MS/MS. n.d., not detected.

10.7717/peerj.20305/supp-5 Supplemental Information 5 Identification of DAAs in different groups.

10.7717/peerj.20305/supp-6 Supplemental Information 6 Identification of anthocyanins contents (μg/g) in coffee samples.

10.7717/peerj.20305/supp-7 Supplemental Information 7 Identification of carotenoid metabolite contents (μg/g) in coffee samples based on LC-MS/MS. n.d., not detected.

10.7717/peerj.20305/supp-8 Supplemental Information 8 Identification of DACs in different groups.

10.7717/peerj.20305/supp-9 Supplemental Information 9 Identification of carotenoids contents (μg/g) in coffee samples.

10.7717/peerj.20305/supp-10 Supplemental Information 10 Statistic Reads of RNA-Seq data.

10.7717/peerj.20305/supp-11 Supplemental Information 11 Statistics of up-regulated and down-regulated DEGs.

10.7717/peerj.20305/supp-12 Supplemental Information 12 Transcription expression of genes involved in anthocyanin biosynthesis.

10.7717/peerj.20305/supp-13 Supplemental Information 13 Transcription expression of genes involved in carotenoid biosynthesis.

10.7717/peerj.20305/supp-14 Supplemental Information 14 Raw data for qRT-PCR measurements.

10.7717/peerj.20305/supp-15 Supplemental Information 15 MIQE checklist.

## Additional Information and Declarations

### DNA Deposition

The following information was supplied regarding the deposition of DNA sequences:

The raw sequence data are available at China National Center for Bioinformation NGDC BioProject: PRJCA028798.

### Data Availability

The following information was supplied regarding data availability:

The data is available in the Supplemental Files.

The raw sequence data are available at China National Center for Bioinformation NGDC BioProject: PRJCA028798.

The clean reads were mapped to the *C. arabica* L. reference genome: https://www.ncbi.nlm.nih.gov/datasets/genome/GCF_003713225.1/.
